# Gestational TSH and FT4 Reference Intervals in Chinese Women: A Systematic Review and Meta-Analysis

**DOI:** 10.3389/fendo.2018.00432

**Published:** 2018-08-03

**Authors:** Xiaotong Gao, Yongze Li, Jiashu Li, Aihua Liu, Wei Sun, Weiping Teng, Zhongyan Shan

**Affiliations:** ^1^Department of Endocrinology and Metabolism, Institute of Endocrinology, Liaoning Provincial Key Laboratory of Endocrine Diseases, The First Affiliated Hospital of China Medical University, China Medical University, Shenyang, China; ^2^Department of Thyroid Surgery, The First Affiliated Hospital of China Medical University, China Medical University, Shenyang, China

**Keywords:** TSH, FT4, pregnancy, reference range, Chinese women

## Abstract

**Background:** Serum thyroid-stimulating hormone (TSH) and free thyroxine (FT4) change dynamically during pregnancy. Differences in geographic regions, populations, and manufacturer's methodologies can affect the reference intervals for thyroid function tests. The 2017 guidelines of the American Thyroid Association (ATA) recommended 4.0 mU/L as the cut-off point for the upper limit of serum TSH in early pregnancy. A systematic review is called for to establish practical, gestational-specific TSH and FT4 reference intervals for pregnant Chinese women and to explore whether the criteria are suitable for China.

**Methods:** English and Chinese articles published from inception to Aug 2017 were searched in the PubMed, EMBASE, and SCIE English-language databases and the CNKI, WanFang, and CQVIP Chinese databases. The relative descent or ascent rates of serum TSH and FT4 were calculated, after which Comprehensive Meta-Analysis V2.0 software was used to analyze the data.

**Results:** Eleven studies (6 in English and 5 in Chinese), five kits and 11,629 Chinese women from nine cities were considered in this meta-analysis. Compared with the reference ranges provided by manufacturers, serum TSH decreased in the first trimester, with the upper limit declining by 21.7% (5.0–36.6%), to a value close to 4.0 mU/L, and the lower limit declining by 85.7% (73.5–97.1%). It continued decreasing in the second trimester, with the upper limit declining by 24.0% (6.4–40.9%) and the lower limit declining by 40.7% (9.0–85.7%). For FT4, the upper limit fluctuated slightly, and the lower limit increased by 6.8% (1.0–14.6%) in the first trimester. Serum FT4 dropped gradually, with the upper limit declining by 21.8% (2.5–31.8%) and the lower limit declining by 12.7% (2.6–19.6%) in the second trimester. During the third trimester, the upper limit decreased by 25.1% (12.7–35.0%), while the lower limit decreased by 20.9% (14.8–27.3%).

**Conclusions:** Various regions, kits and test methods affect the gestational TSH and FT4 levels. The non-pregnant serum TSH upper limit minus 22% is very close to 4.0 mU/L, which can be used as a sub-optimal approach to represent the cut-off value for pregnant Chinese women in the first trimester.

## Introduction

Thyroid hormone is essential for the growth and development of the human body. It plays a vital role in promoting the development of the skeletal, nervous, and reproductive systems (1). Pregnancy affects the thyroid gland and its function profoundly. Human chorionic gonadotrophin (hCG) significantly increases in early pregnancy, sharing the same alpha-subunits and 80%-homologous beta-subunits with TSH. Therefore, hCG can stimulate thyroid hormonogenesis, which is the negative-feedback system to TSH secretion, causing the serum TSH level to decline during early pregnancy (2, 3).

Serum TSH and FT4 vary with gestational age. Several studies and guidelines have indicated that non-pregnant reference intervals of serum TSH and FT4 are not applicable for diagnosing thyroid diseases during pregnancy. By contrast, trimester- and method-specific reference ranges for thyroid testing have been strongly recommended because of their higher accuracy (3–6). Nevertheless, the formulation of gestational reference ranges is affected by many factors, limiting their feasibility (7, 8). The 2011 guidelines of the American Thyroid Association (ATA) suggested a specific upper limit cut-off (2.5 mU/L) for serum TSH in the first trimester of pregnancy (4). However, there are large differences in TSH and FT4 reference ranges between various populations, with 90% of the relevant studies having higher TSH upper limits than the TSH cut-off point of 2.5 mU/L. These inconsistencies could increase the misdiagnosis rate of overt and subclinical hypothyroidism in pregnancy (9). The 2017 ATA guidelines noted that if internal or transferable pregnancy-specific TSH reference intervals are unavailable, an upper reference limit of 4.0 mU/L may be used, representing the non-pregnant TSH upper limit minus 0.5 mU/L (5). However, it is uncertain whether this cut-off is appropriate for pregnant Chinese women. Similarly, gestational- and method-specific criteria are also recommended for serum FT4 (5), but the criteria for serum FT4 are as inconvenient as those of TSH to diagnose gestational hypothyroxinemia in clinical practice.

The aim of the current study was to systematically assess and summarize gestational- and method-specific serum TSH and FT4 reference ranges in various regions in China and determine their trends in early, middle and late pregnancy. We compared the differences between the reference ranges of Chinese pregnant women and the 2017 ATA guidelines recommendation of 4.0 mU/L. Finally, we aimed to provide feasible and practical reference intervals to diagnose hypothyroidism and hypothyroxinemia in pregnancy.

## Materials and methods

### Search strategy and selection criteria

A systematic literature search (PubMed, EMBASE, SCIE, Chinese National Knowledge Infrastructure, Chinese Scientific Journals Full-text Database, Wanfang) was performed from inception to Aug 2017. The keywords “TSH” and “FT4” combined with the terms “reference range” or “reference interval,” “pregnant,” or “gestational,” and “China” or “Chinese” were used to search for potentially relevant studies in English and Chinese. The following is an example for PubMed: (((((#TSH AND #FT4))) AND ((#pregan^*^ OR #gestation^*^)) AND ((#China OR #Chinese)) AND ((#reference range^*^ OR #reference interval^*^)). To identify additional studies and expand our search, the reference lists of the retrieved articles were scanned.

The studies included in the meta-analysis conformed with the following conditions: All subjects were pregnant Chinese women. The study recruitment standards met the National Academy of Clinical Biochemistry (NACB) recommendations: (1) more than 120 subjects; (2) no TPOAb or TGAb positivity; (3) no family or personal history of thyroid disease; (4) no goiter; and (5) no medical history influencing thyroid function (except use of estrogens) (6).

Exclusion criteria were as follows: (1) subjects came from iodine-excessive or iodine-deficient regions; (2) the Newcastle-Ottawa quality assessment scale (NOS) quality score was <6 (10); (3) serum TSH- and FT4-related information could not be extracted; and (4) the study was a repeat of an earlier study. In addition, Li et al. (11) declared that the reference intervals for non-pregnant women should be used from 4 to 6 gestational weeks. Therefore, studies including 0–6 gestational weeks or average gestational week <9.3 weeks in the first trimester were excluded to improve the accuracy of the meta-analysis.

### Quality assessment

The NOS was selected to assess the quality of the included studies using the “star system.” Information regarding selection, comparability, and outcomes was evaluated with a maximum of 4 stars, 2 stars, and 3 stars, respectively. The total full score = 9. A study graded ≥6 stars was considered a high-quality study (10).

### Data extraction

Two reviewers (Gao XT and Li YZ) abstracted the following data from all eligible studies independently: first author; publication year and journal; region(s) and hospital(s) of study; sample size; pregnancy stages; medians and percentiles (2.5^th^ and 97.5^th^) of serum TSH and FT4; manufacturers; inter- and intra-assay coefficients of variation (CV) in the laboratory; normal range of the detection kit; normal range of the control group; and iodine status of the region.

### Statistical analysis

We summarized the lower reference limits (2.5^th^) and the upper reference limits (97.5^th^) of serum TSH and FT4 in early, middle and late pregnancy. We calculated the relative descent or ascent rate of serum TSH and FT4 and compared these with the normal reference ranges provided by manufacturers involved in each enrolled study. The calculation formula can be written as:

1. Relative descent rate of lower limit = (2.5^th^ in non-pregnancy−2.5^th^ in pregnancy)/2.5^th^ in non-pregnancy × 100%;
2. Relative descent rate of upper limit = (97.5^th^ non-pregnancy−97.5^th^ in pregnancy)/97.5^th^ non-pregnancy × 100%.

The meta-analysis of the relative descent and ascent rates for the gestational reference intervals was accomplished using Comprehensive Meta-Analysis software (V2.0, Biostat, Englewood, NJ). The Z test was used to compare the difference between 0 and the relative change rates of TSH and FT4 reference intervals (*p* < 0.05, 0.05 < *p* < 0.1 and *p* > 0.1 indicated high, medium, and no difference between relative change rate and 0, respectively).

Factors affecting gestational TSH and FT4 were age, iodine nutrition status, ethnicity, sex, and hour of the day, in addition to the conditions referred to in the NACB (12). Our meta-analysis included pregnant women of appropriate age who came from adequate-iodine regions of China, and the blood samples were taken in morning in the fasting state.

## Results

### Literature search and study characteristics

**Figure 1:**
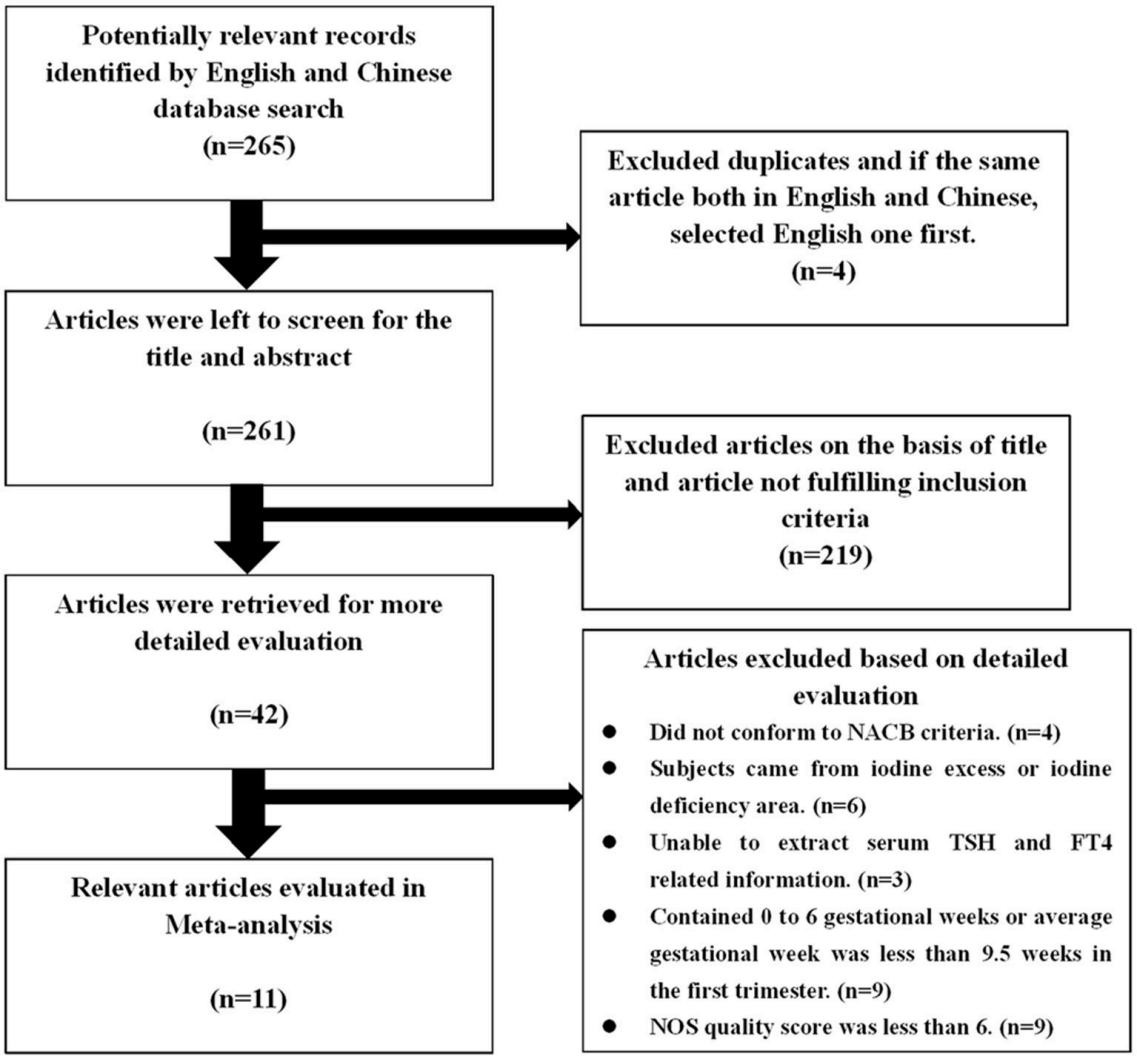
Flow chart of the study selection process.

A total of 265 studies were initially considered for inclusion, of which 4 were excluded due to duplication, and 219 articles were excluded after screening the titles and abstracts. After more detailed evaluation of the remaining 42 articles, 31 articles were excluded. Finally, the remaining 11 studies (6 published in English and 5 in Chinese) involving 5 types of kits and including 11,629 Chinese women met the inclusion criteria and were included in this meta-analysis (Figure 1). There were 4 studies on the application of Roche e600/601 with 1,920 pregnant Chinese women; 3 studies using the Bayer ADVIA Centaur with 3,441 pregnant Chinese women; 4 studies using the Beckman with 2,350 pregnant Chinese women; 2 studies using the Abbott Architect I 2,000 with 1,223 pregnant Chinese women, and 2 studies using the DPC Immulite 1,000 with 1,189 pregnant Chinese women. The qualified studies were published from 2008 to 2016 and proved to be of good quality in accordance with the NOS scoring system (Supplementary Table 1).

### Gestational-specific serum TSH and FT4 alterations

**Figure 2:**
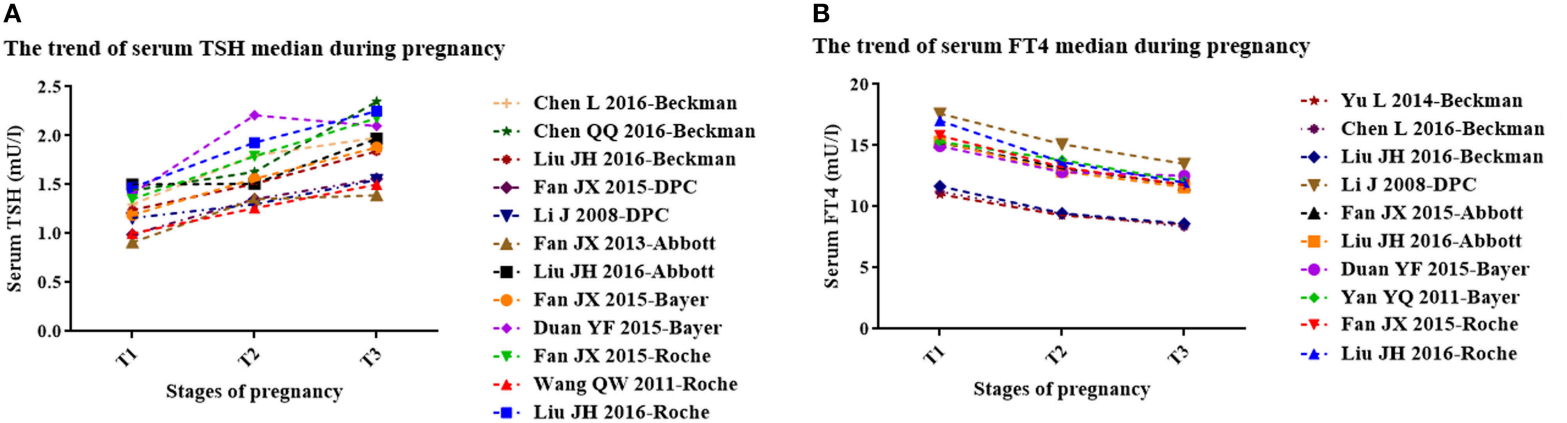
The trend of serum TSH **(A)** and FT4 **(B)** median during pregnancy. *T1*, the first trimester of pregnancy; *T2*, the second trimester of pregnancy; *T3*, the third trimester of pregnancy. **(A)** The trend of median gestational serum TSH in each study (3 studies with Roche kits; 2 studies with Bayer kits; 2 studies with Abbott kits; 2 studies with DPC kits; 3 studies with Beckman kits). **(B)** The trend of gestational serum FT4 median in each study (2 studies with Roche kits; 2 studies with Bayer kits; 2 studies with Abbott kits; 1 study with DPC kits; 3 studies with Beckman kits).

Table 1 displays the basic characteristics of the included studies regarding serum TSH. According to the median, serum TSH decreased in early pregnancy and showed an upward trend during middle and late pregnancy (Figure 2A).

**Table 1:** Gestational TSH reference intervals and relative descent or ascent rate compared with non-pregnancy in Chinese women.

**Manufacturer**	**References**	**Location**	**Gestational weeks, samples**	**Median, percentiles (2.5th and 97.5th), mU/L**			**Relative descent rate in the first, second, third trimesters of pregnancy, %**					
				**T1**	**T2**	**T3**	**^c^ T1, 2.5th**	**^c^ T2, 2.5th**	**^c^ T3, 2.5th**	**^c^ T1, 97.5th**	**^c^ T2, 97.5th**	**^c^ T3, 97.5th**
RocheE600/601 0.69–5.64^a^mU/L	Liu et al. (28)	Shenyang	T1 (8–12wk): 144 T2 (12–27wk): 304 T3 (27–40wk): 331	1.47 (0.09–4.52)	1.93 (0.45–4.32)	2.25 (0.71–5.46)	86.96	34.78	−2.90	19.86	23.40	3.19
	Wang et al. (29)	Changzhou	T1 (10–14wk): 301 T2 (20–24wk): 301 T3 (30–34wk): 301	1.00 (0.02–3.65)	1.26 (0.36–3.46)	1.5 (0.44–5.04)	97.10	47.83	36.23	35.28	38.65	10.64
	Fan et al. (30)	Shanghai	T1 (9–12wk): 200 T2 (16–24wk): 200 T3 (32–36wk): 200	1.35 (0.08–4.12)	1.79 (0.43–4.04)	2.18 (0.67–5.65)	88.41	37.68	2.90	27.08	28.37	−0.18
	Li et al. (11)	Shenyang	T1 (7–12wk): 640	1.47 (0.10–4.34)	–	–	85.51	–	–	23.05	–	–
BayerADVIA Centaur 0.55–4.78^b^mU/L	Duan et al. (31)	Sichuan	T1 (10–14wk): 963 T2 (20–24wk): 981 T3 (30–34wk): 792	1.41 (0.05–4.49)	2.21 (0.61–4.97)	2.10 (0.65–4.63)	92.54	8.96	2.99	15.44	6.40	12.81
	Fan et al. (30)	Shanghai	T1 (9–12wk): 200 T2 (16–24wk): 200 T3 (32–36wk): 200	1.19 (0.07–3.38)	1.56 (0.33–3.34)	1.88 (0.59–4.88)	87.27	40.00	−7.27	29.29	30.13	−2.09
AbbottArchitectI 20000.35–4.94^b^ mU/L	Liu et al. (28)	Shenyang	T1 (8–12wk): 144 T2 (12–27wk): 304 T3 (27–40wk): 331	1.50 (0.03–3.83)	1.51 (0.05–3.71)	1.97 (0.47–6.29)	91.43	85.71	−34.29	22.47	24.90	−27.33
	Fan et al. (32)	Shanghai	T1 (9–13wk): 140 T2 (16–28wk): 184 T3 (28–40wk): 120	0.91 (0.03–3.60)	1.35 (0.14–3.61)	1.39 (0.17–3.59)	91.43	60.00	51.43	27.13	26.92	27.33
DPCImmulite 1000 0.40–4.00^b^mU/L	Li et al. (33)	Shenyang	T1 (8–12wk): 249 T2 (13–24wk): 375 T3 (24–40wk): 365	1.16 (0.09–3.8)	1.30 (0.26–3.50)	1.55 (0.42–3.85)	77.50	35.00	−5.00	5.00	12.50	3.75
	Xu et al. (30)	Shanghai	T1 (9–12wk): 200 T2 (16–24wk): 200 T3 (32–36wk): 200	0.99 (0.08–3.00)	1.35 (0.31–2.97)	1.56 (0.49–4.95)	80.00	22.50	−22.50	25.00	25.75	−23.75
BeckmanUniCel DX I 800 0.34–5.60^b^mU/L	Liu et al. (28)	Shenyang	T1 (8–12wk): 144 T2 (12–27wk): 304 T3 (27–40wk): 331	1.24 (0.05–3.55)	1.51 (0.21–3.31)	1.84 (0.62–5.06)	85.29	38.24	−82.35	36.61	40.89	9.64
	Chen and Wang (34)	Zhejiang	T1 (9–12wk): 281 T2 (16–24wk): 281 T3 (12–36wk): 281	1.44 (0.05–3.97)	1.63 (0.12 −4.28)	2.35 (0.30–6.01)	85.29	64.71	11.76	29.11	23.57	−7.32
	Chen et al. (35)	Chongqing	T1 (10–13wk+6): 303 T2 (14–27wk+6): 158 T3 (30–34wk): 132	1.3 (0.09–4.85)	1.80 (0.11–5.13)	1.98 (0.75–3.67)	73.53	67.65	−120.59	13.21	8.39	34.46

T1, the first trimester of pregnancy; T2, the second trimester of pregnancy; T3, the third trimester of pregnancy; 2.5th, the lower reference limit; 97.5th, the upper reference limit; TSH, thyroid stimulating hormone.

a *The TSH reference range provided by Roche was 0.27–4.20 mU/L, lower than the reference ranges tested for normal populations in included studies: 0.51–5.40 mU/L in Liu et al. (28), 0.75–5.28 mU/L in Wang et al. (29), and 0.69–5.64 mU/L in Li et al. (11), respectively. This suggested that the reference range provided by Roche was not suitable for Chinese populations. In our study, 0.69–5.64 mU/L in Li et al. (11) was used as the non-pregnant reference range for Roche*.

b *Normal serum TSH reference range provided by manufactures*.

c T1, 2.5th means the relative descent rate of serum TSH lower limit in the first trimester of pregnancy. The calculation formula can be written as: *(2.5th in non-pregnancy−2.5th in pregnancy)/2.5th in non-pregnancy × 100%. The same formula was applied in the second and third trimesters of pregnancy*. T1, 97.5th means the relative descent rate of serum TSH upper limit in the first trimester of pregnancy. The calculation formula can be written as: *(97.5th in non-pregnancy−97.5th in pregnancy)/97.5th in non-pregnancy × 100%. The same formula was applied in the second and third trimesters of pregnancy*.

Table 2 shows the basic characteristics of the included studies regarding serum FT4. In the first trimester, no obvious rule was derived for the serum FT4 upper limit (lower than the non-pregnant levels in six studies; higher than the non-pregnant levels in the other five studies). However, the lower limits were higher than those in non-pregnancy. In the second and third trimesters, both the upper and lower limits of serum FT4 were lower than those in non-pregnancy. The gestational serum FT4 medians exhibited a downward trend (Figure 2B).

**Table 2:** Gestational FT4 reference intervals and relative descent or ascent rate compared with non-pregnancy in Chinese women.

**Manufacturer**	**First author, published year**	**Location**	**Gestational weeks, samples**	**Median, percentiles (2.5th and 97.5th), pmol/L**			**Relative descent rate in the first, second, third trimesters of pregnancy, %**					
				**T1**	**T2**	**T3**	**^c^ T1, 2.5th**	**^c^ T2, 2.5th**	**^c^ T3, 2.5th**	**^c^ T1, 97.5th**	**^c^ T2, 97.5th**	**^c^ T3, 97.5th**
RocheE600/601 12.00–22.00^a^ pmol/L	Liu et al. (28)	Shenyang	T1 (8–12wk): 144 T2 (12–27wk): 304 T3 (27–40wk): 331	17.02 (13.15–20.78)	13.64 (9.77–18.89)	11.97 (8.72–15.37)	−9.58	18.58	27.33	5.55	14.14	30.14
	Li et al. (11)	Shanghai	T1 (9–12wk): 200 T2 (16–24wk): 200 T3 (32–36wk): 200	15.82 (12.90–19.88)	13.23 (10.40–15.91)	11.77 (9.46–14.31)	−7.50	13.33	21.17	9.64	27.68	34.95
	Li et al. (11)	Shenyang	T1 (7–12wk): 640	15.80 (12.30–20.88)	–	–	−2.50	–	–	5.09	–	–
BayerADVIA Centaur 11.48–22.70^a^ pmol/L	Yan et al. (36)	Tianjin + Beijing	T1 9.5 (5–12wk): 168 T2 (13–27wk): 168 T3 (28–41wk): 169	15.30 (11.80–21.0)	13.80 (10.60–17.60)	12.10 (9.20–16.70)	−2.61	7.83	20.00	7.49	22.47	26.43
	Duan et al. (31)	Sichuan	T1 (10–14wk): 963 T2 (20–24wk): 981 T3 (30–34wk): 792	14.96 (12.29–18.92)	12.82 (10.97–15.49)	12.53 (9.49–16.25)	−7.06	4.44	17.33	16.65	31.76	28.41
Abbott Architect I 2000 12.25–18.87^b^ pmol/L	Liu et al. (28)	Shenyang	T1 (8–12wk): 144 T2 (12–27wk): 304 T3 (27–40wk): 331	15.30 (12.37–19.09)	12.90 (9.85–18.05)	11.59 (9.12–14.91)	−0.98	19.59	25.55	−0.05	4.35	20.99
	Fan et al. (32)	Shanghai	T1 (9–12wk): 200 T2 (16–24wk): 200 T3 (32–36wk): 200	15.25 (12.77–18.55)	13.13 (10.49–15.30)	11.79 (9.57–14.28)	−4.24	14.37	21.88	1.70	18.92	24.32
DPC Immulite1000 11.5–22.7^a^pmol/L	Li et al. (33)	Shenyang	T1 (8–12wk): 249 T2 (13–24wk): 375 T3 (24–40wk): 365	17.60 (12.00–23.34)	15.1 (11.20–21.46)	13.5 (9.80–18.20)	−4.35	2.61	14.78	−2.82	5.46	19.82
BeckmanUniCel DX I 800 7.86–14.61^a^ pmol/L	Chen et al. (35)	Shenyang	T1 (8–12wk): 144 T2 (12–27wk): 304 T3 (27–40wk): 331	11.67 (9.01–15.89)	9.46 (6.62–13.51)	8.61 (5.88–12.76)	−14.63	15.78	25.19	−8.76	7.53	12.66
	Chen et al. (35)	Chongqing	T1 (10–13wk+6): 303 T2 (14–27wk+6): 158 T3 (30–34wk): 132	11.24 (8.42–15.75)	9.43 (6.50–14.24)	8.37 (6.12–11.69)	−7.12	17.30	22.14	−7.80	2.53	19.99
	Yu et al. (37)	Shenzhen	T1 (10–13W): 334 T2 (14–26W): 272 T3 (27–42W): 271	11.01 (8.52–14.68)	9.29 (6.84–11.91)	8.55 (6.65–10.96)	−8.40	12.98	15.39	−0.48	18.48	24.98

T1, the first trimester of pregnancy; T2, the second trimester of pregnancy; T3, the third trimester of pregnancy; 2.5th, the lower reference limit; 97.5th, the upper reference limit; FT4, free T4.

a *Normal serum FT4 reference range provided by manufactures*.

b *Abbott offered a FT4 reference range of 9.01–19.05 pmol/L, which lower limit was little than the gestational lower limits in Liu et al. (28) of 12.37 pmol/L and Fan et al. (32) of 12.77 pmol/L. Moreover, it also lower than the lower limit of normal population provided by Liu et al. (28) of 12.25–18.87 pmol/L. Thus, the reference range offered by Abbott was not suitable for Chinese population, and we use 12.25–18.87 pmol/L as the non-pregnant reference range for Abbott*.

c T1, 2.5th means the relative descent rate of serum FT4 lower limit in the first trimester of pregnancy. The calculation formula can be written as: *(2.5th in non-pregnancy−2.5th in pregnancy)/2.5th in non-pregnancy × 100%. The same formula was applied in the second and third trimesters of pregnancy*. *T1, 97.5th means the relative descent rate of serum FT4 upper limit in the first trimester of pregnancy. The calculation formula can be written as*: *(97.5th in n non-pregnancy−97.5th in pregnancy)/97.5th in non-pregnancy × 100%. The same formula was applied in the second and third trimesters of pregnancy*.

We used the random-effects model to summarize the descending rule of serum TSH in early pregnancy and the descending and ascending rules of serum FT4 in each gestational stage by meta-analysis.

### Comparison of the serum TSH upper and lower limits between pregnancy and non-pregnancy

#### Variations in the serum TSH reference ranges in early pregnancy

**Figure 3:**
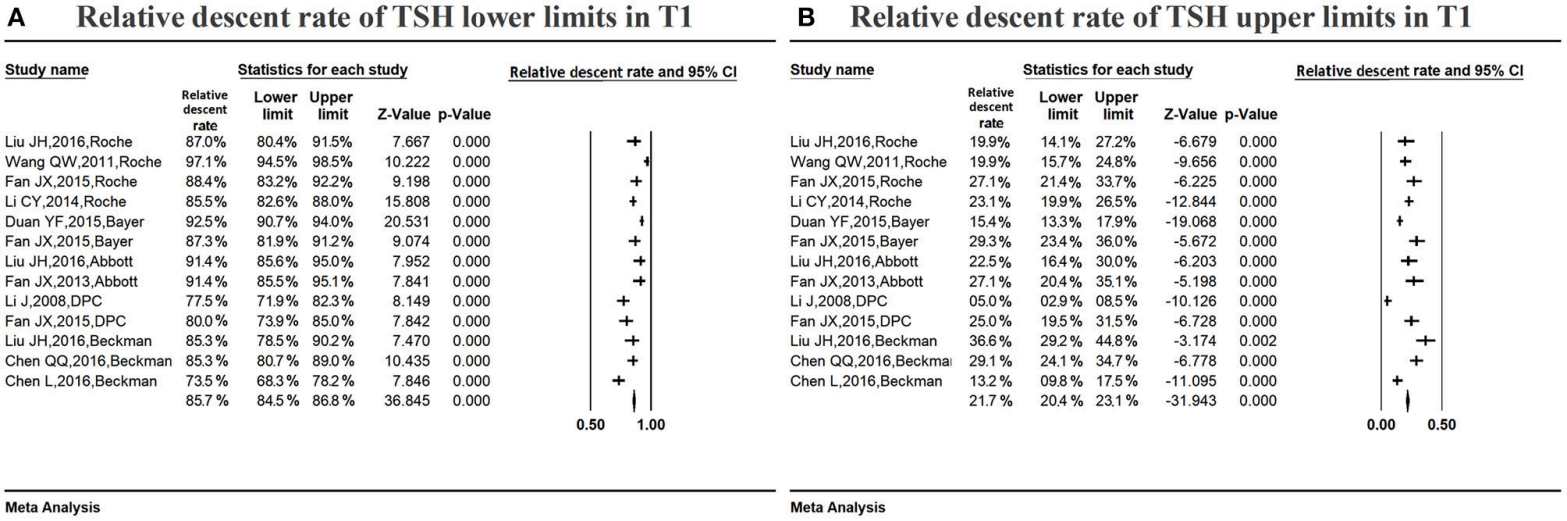
Meta-analysis of the relative descent rate of TSH lower **(A)** and upper **(B)** reference limits in early pregnancy. Figure shows unadjusted relative descent rate of TSH lower **(A)** and upper **(B)** limit estimates in early pregnancy with 95% confidence limits for each study selected. Pooled relative descent rate estimates are represented as diamonds in this plot.

Figure 3A shows the summarized relative descent rate [85.7%, 95% confidence interval (CI): 84.5, 86.8%] for the serum TSH lower limit in the first trimester from 2008 to 2016. The relative descent rate in each study ranged from 73.5% (95% CI: 68.3, 78.2%) to 97.1% (95% CI: 94.5, 98.5%). This suggested that the lower limit of serum TSH decreased in the first trimester compared with that in non-pregnancy, and the descent rate was 85.7% (73.5–97.1%).

Figure 3B shows the summarized relative descent rate (21.7%, 95% CI: 20.4, 23.1%) for the serum TSH upper limit in the first trimester. The relative descent rate in individual studies ranged from 5.0% (95% CI: 2.9, 8.5%) to 36.6% (95% CI: 29.2, 44.8%), suggesting that, compared to the non-pregnant levels, the serum TSH upper limit decreased in early pregnancy, and the descent rate was 21.7% (5.0–36.6%).

#### Comparison of serum TSH upper reference limits under different conditions

**Figure 4:**
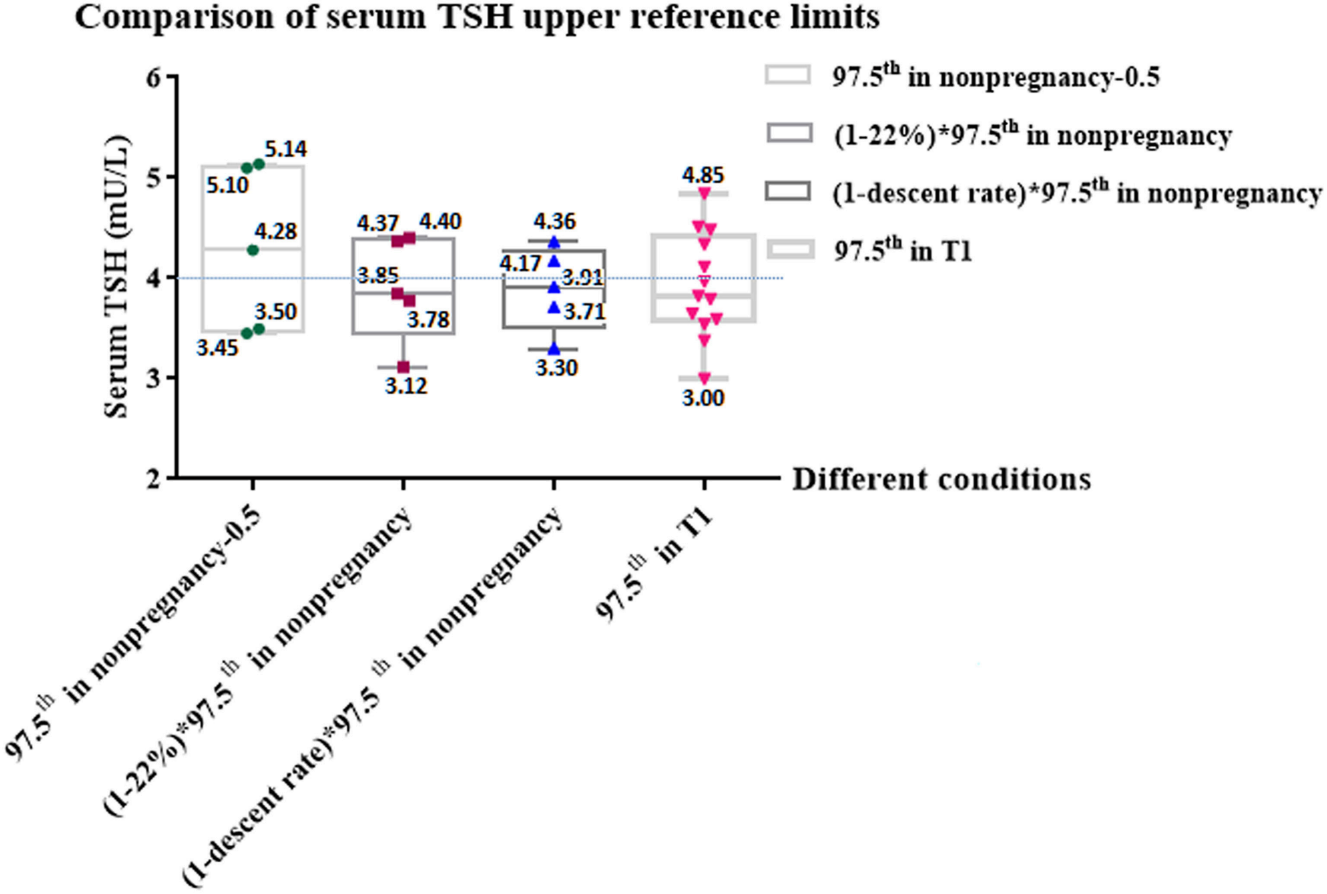
Comparison of serum TSH upper reference limits under different conditions. Figure shows the comparison among serum TSH upper reference limits acquired in different conditions and shows the gaps between the limits and 4.0 mU/L. *T1*, the first trimester of pregnancy; *97.5th*, the upper reference limit; *TSH*, thyroid stimulating hormone; *97.5th in non-pregnancy*, the normal TSH upper limits provided by 5 kinds of manufacturers (Roche, Bayer, Abbott, DPC, and Beckman); *97.5th in T1*, The real serum TSH upper limits during early pregnancy provided by 11 enrolled studies. *97.5th in non-pregnancy*–*0.5*, We subtracted 0.5 mU/L from the normal TSH upper limits provided by 5 kinds of manufacturers (Roche, Bayer, Abbott, DPC, and Beckman), according to 2017 ATA guidelines' recommendation that 4.0 mU/L represents a reduction in the non-pregnant TSH upper reference limit of ~0.5 mU/L (5); *(1*–*22%)* × *97.5th in non-pregnancy*, In our meta-analysis, regardless of manufacture, the non-pregnant serum TSH upper limit decreased by 22% in the first trimester of pregnancy; *(1*–*descent rate)* × *97.5th in non-pregnancy*, In our meta-analysis, the relative descent rate of the TSH upper limit during early pregnancy assayed by Roche, Bayer, Abbott, DPC and Beckman were 22.7, 18.3, 24.8, 17.6, and 25.5%, respectively. The relative descent rate of each kit is listed in Supplementary Table 2. We calculated the method-specific gestational upper limit by non-pregnant upper limit decreased by the relative descent rate.

Figure 4 shows the comparison of the serum TSH upper limits acquired in different conditions. If we subtract 0.5 mU/L from the upper limits provided by manufacturers (97.5th in non-pregnancy), the gestational TSH upper limits obtained (97.5th in non-pregnancy−0.5), which ranged from 3.45 to 5.14 mU/L, varied greatly, and the gaps around 4.0 mU/L, which ranged from −0.55 to 1.14 mU/L, were different from each other. The absolute values of the gaps were >1, suggesting that the fluctuation around 4.0 mU/L was obvious.

By contrast, if we compare 4.0 mU/L with the gestational TSH upper limit, which was 22% lower than the non-pregnant upper limit, [(1–22%) × 97.5th in non-pregnancy], ranging from 3.12 to 4.40 mU/L, the gaps ranging from −0.88 to 0.40 mU/L were narrower than those of “97.5th in non-pregnancy – 0.5” ranging from −0.55 to 1.14 mU/L. Similarly, if we replace 22% with the relative descent rate of each kit (Roche, Bayer, Abbott, DPC and Beckman were 22.7, 18.3, 24.8, 17.6 and 25.5%, respectively) listed in Supplementary Table 2, the gestational upper limits obtained [(1–descent rate) × 97.5th in non-pregnancy] ranged from 3.30 to 4.36 mU/L. The gaps between 4.0 mU/L and ‘(1–descent rate) × 97.5^th^ in non-pregnancy' were much narrower, which ranged from −0.70 to 0.36 mU/L. The absolute values of the gaps in both groups were less than 1, suggesting that the non-pregnant upper limit that declined by its relative descent rate was much closer to 4.0 mU/L.

Figure 4 also shows that the comparison between 4.0 mU/L and the TSH upper limits of the first trimester in Chinese women (97.5^th^ in T1). If we subtract “97.5^th^ in T1” from 4.0 mU/L, the gaps ranged from −0.85 to 1.0 mU/L. The absolute values of the gaps were ≤1. These results suggest that regardless of efforts to standardize the reference ranges, there were still differences in comparison to the real TSH upper limits of pregnant Chinese women, while the differences were not very significant.

#### Variations in the serum TSH reference ranges in middle pregnancy

**Figure 5:**
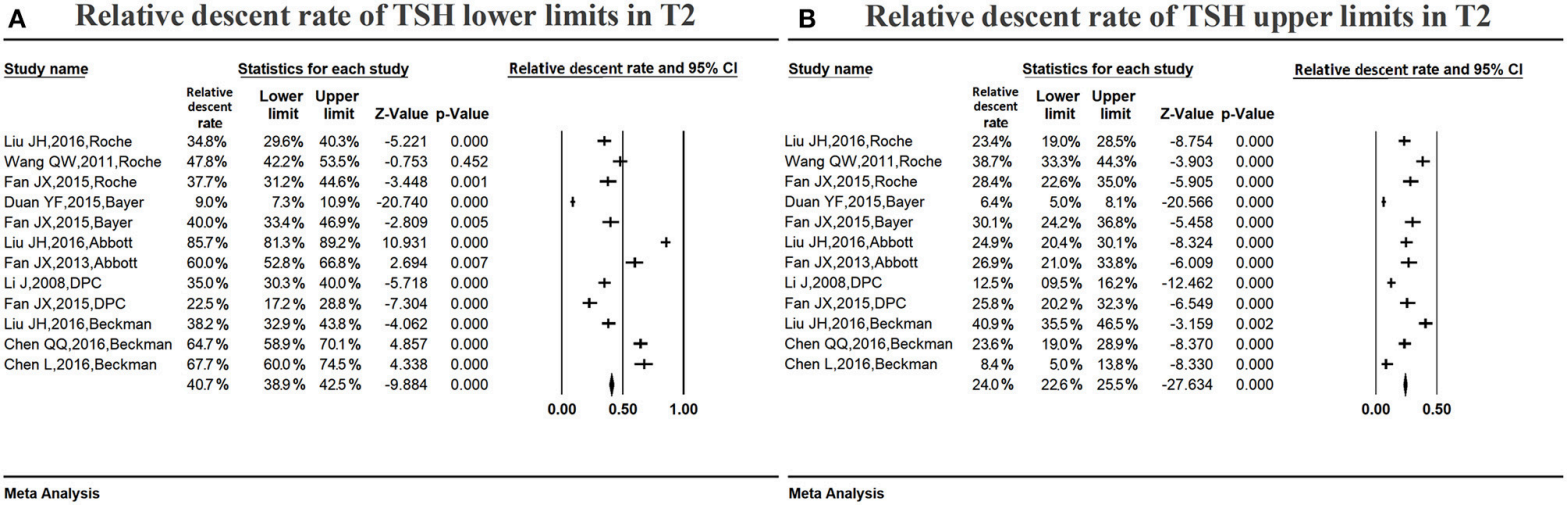
Meta-analysis of relative descent rate of TSH lower **(A)** and upper **(B)** reference limits in middle pregnancy. Figure shows unadjusted relative descent rate of TSH lower **(A)** and upper **(B)** limit estimates in middle pregnancy with 95% confidence limits for each study selected. Pooled relative descent rate estimates are represented as diamonds in this plot.

Figure 5A shows the summarized relative descent rate (40.7%, 95% CI: 38.9, 42.5%) for the serum TSH lower limit in the second trimester. The relative descent rate in each study ranged from 9.0% (95% CI: 7.3, 10.9%) to 85.7% (95% CI: 81.3, 89.2%). This suggests that the lower limit of serum TSH decreased in the second trimester compared with that in non-pregnancy, and the descent rate was 40.7% (9.0–85.7%).

Figure 5B shows the summarized relative descent rate (24.0%, 95% CI: 22.6, 25.5%) for the serum TSH upper limit in the second trimester. The relative descent rate in individual studies ranged from 6.4% (95% CI: 5.0, 8.1%) to 40.9% (95% CI: 35.5, 46.5%), suggesting that, compared to the non-pregnant levels, the serum TSH upper limit decreased in middle pregnancy, and the descent rate was 24.0% (6.4–40.9%).

#### Variations in the serum TSH reference ranges in late pregnancy

Table 1 lists the changing characteristics of the serum TSH lower limit in the third trimester. Seven studies showed that the lower limit increased compared with non-pregnant levels. By contrast, the lower limit decreased in the other 5 studies. The fluctuation range varied from down by 51.43% to up by 120.59%. Therefore, there was no definite change rule regarding the TSH lower limit in late pregnancy, and the fluctuation range was wide.

Table 1 also lists the changing characteristics of the serum TSH upper limit in late pregnancy. Five studies showed that the upper limit increased compared with non-pregnant levels. By contrast, the upper limit decreased in the other 7 studies. The fluctuation range varied from down by 34.46% to up by 27.33%. Therefore, there was no definite change rule regarding the TSH upper limit in the third trimester, and the fluctuation range was wide.

### Comparison of the serum FT4 upper and lower limits between pregnancy and non-pregnancy

#### Variations in the serum FT4 reference ranges in early pregnancy

**Figure 6:**
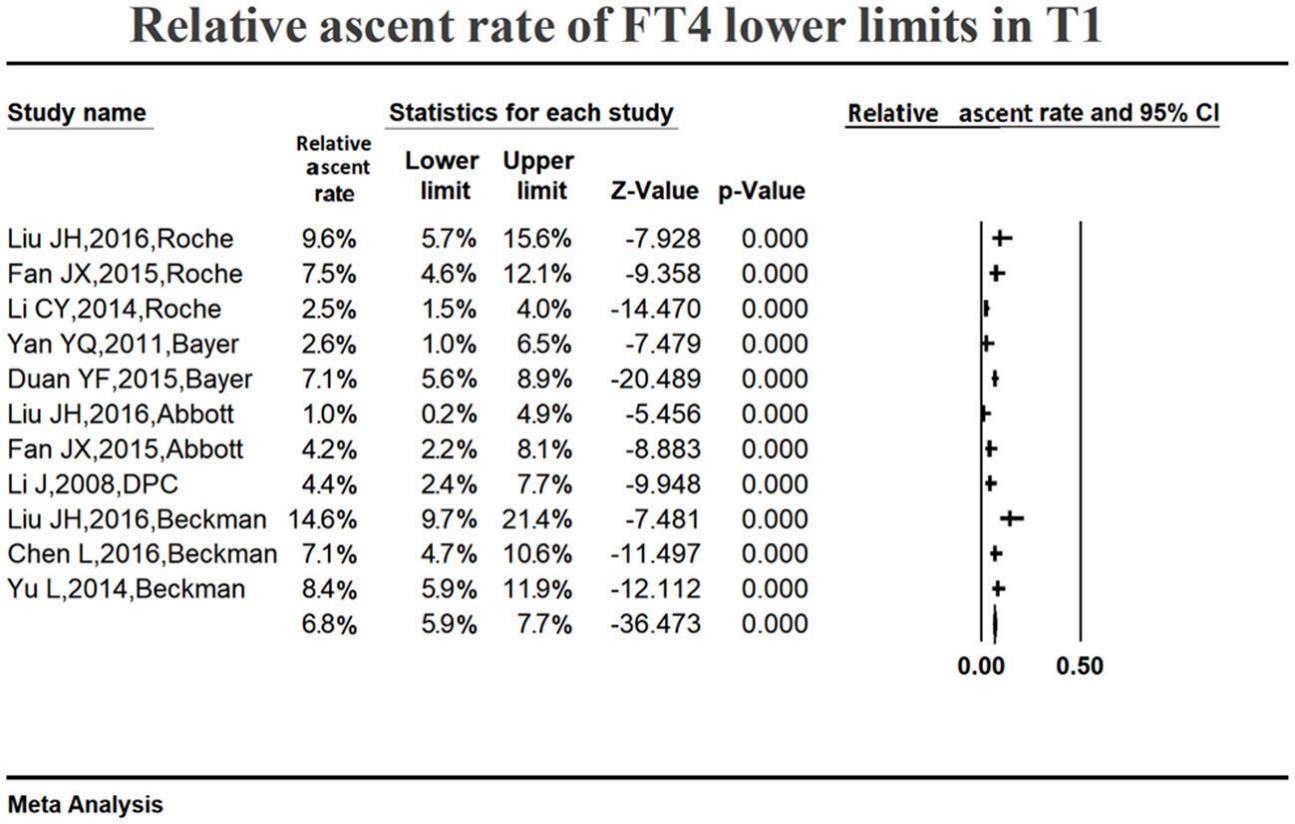
Meta-analysis of relative ascent rate of FT4 lower reference limits in early pregnancy. Figure shows unadjusted relative ascent rate of FT4 lower limit estimates in early pregnancy with 95% confidence limits for each study selected. Pooled relative ascent rate estimates are represented as diamonds in this plot.

Figure 6 shows the summarized relative ascent rate (6.8%, 95% CI: 5.9, 7.7%) for the serum FT4 lower limit in the first trimester. The relative ascent rate in all studies ranged from 1.0% (95% CI: 0.2, 4.9%) to 14.6% (95% CI: 9.7, 21.4%), suggesting that the serum FT4 lower limit increased in early gestation compared to the non-pregnant levels, and the ascent rate was 6.8% (1.0–14.6%).

Table 2 lists the changing characteristics of the serum FT4 upper limit in the first trimester. Six studies showed that the upper limit decreased compared with non-pregnant levels. By contrast, the upper limit increased in the other 5 studies. The fluctuation range varied from down by 16.65% to up by 8.76%. Therefore, there was no definite change rule regarding the FT4 upper limit in early pregnancy, and the fluctuation range was small.

#### Variations in the serum FT4 reference ranges in middle pregnancy

**Figure 7:**
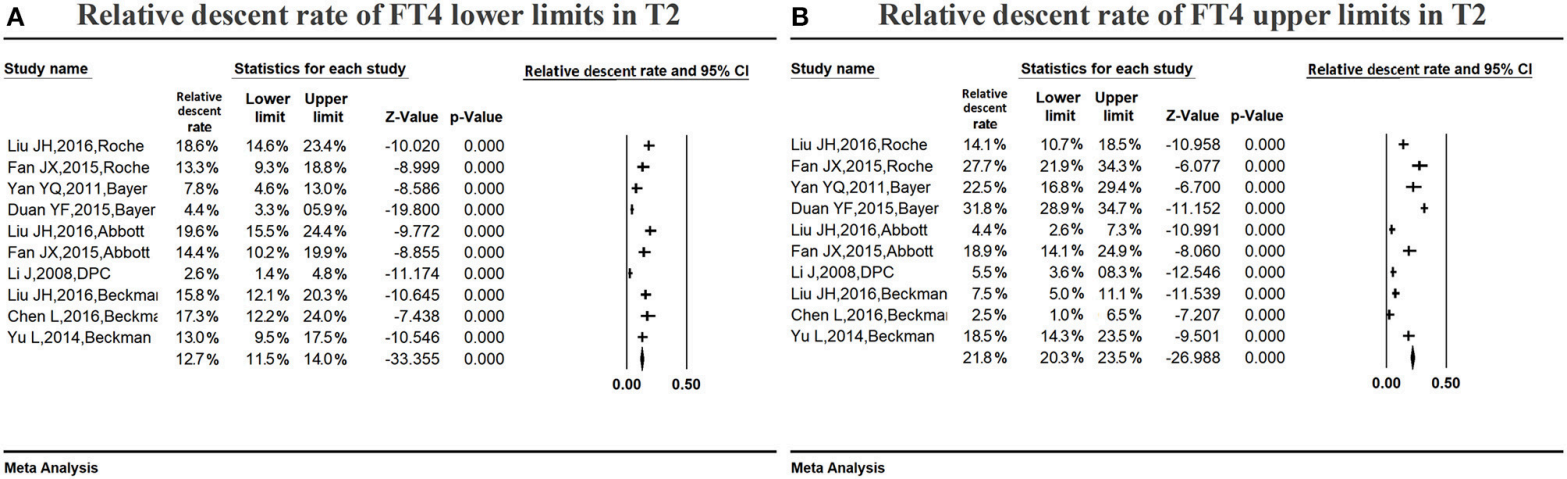
Meta-analysis of relative descent rate of FT4 lower **(A)** and upper **(B)** reference limits in middle pregnancy. Figure shows unadjusted relative descent rate of FT4 lower **(A)** and upper **(B)** limit estimates in middle pregnancy with 95% confidence limits for each study selected. Pooled relative descent rate estimates are represented as diamonds in this plot.

Figure 7A summarizes the relative descent rate (12.7%, 95% CI: 11.5, 14.0%) with regard to the serum FT4 lower limit during the second trimester. The relative descent rate in the included studies ranged from 2.6% (95% CI: 1.4, 4.8%) to 19.6% (95% CI: 15.5, 24.4%). This suggested that the serum FT4 lower limit decreased in middle pregnancy compared with non-pregnant levels, and the descent rate was 12.7% (2.6–19.6%).

Figure 7B summarizes the relative descent rate (21.8%, 95% CI: 20.3, 23.5%) for the serum FT4 upper limit in the second trimester. The relative descent rate in the included studies ranged from 2.5% (95% CI: 1.0, 6.5%) to 31.8% (95% CI: 28.9, 34.7%). This suggested that the serum FT4 upper limit decreased in middle pregnancy compared with the non-pregnant levels, and the descent rate was 21.8% (2.5–31.8%).

#### Variations in the serum FT4 reference ranges in late pregnancy

**Figure 8:**
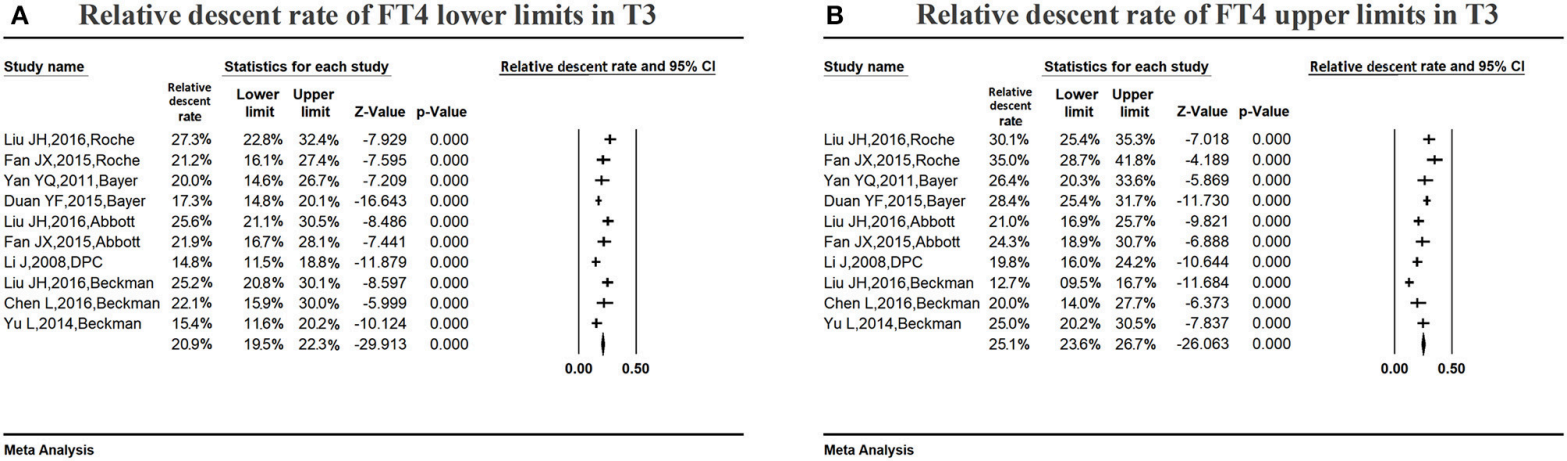
Meta-analysis of relative descent rate of FT4 lower **(A)** and upper **(B)** reference limits in late pregnancy. Figure shows unadjusted relative descent rate of FT4 lower **(A)** and upper **(B)** limit estimates in late pregnancy with 95% confidence limits for each study selected. Pooled relative descent rate estimates are represented as diamonds in this plot.

Figure 8A shows the summarized relative descent rate (20.9%, 95% CI: 19.5, 22.3%) for the serum FT4 lower limit in the third trimester. The relative descent rate in the included studies ranged from 14.8% (95% CI: 11.5, 18.8%) to 27.3% (95% CI: 22.8, 32.4%). This suggested that the lower limit of serum FT4 decreased in the third trimester of pregnancy compared with the non-pregnant levels, and the descent rate was 20.9% (14.8–27.3%).

Figure 8B shows the summarized relative descent rate (25.1%, 95% CI: 23.6, 26.7%) for the serum FT4 upper limit in the third trimester. The relative descent rate in all studies ranged from 12.7% (95% CI: 9.5, 16.7%) to 35.0% (95% CI: 28.7, 41.8%), suggesting that the serum FT4 upper limit declined in late gestation compared with non-pregnant levels, and the descent rate was 25.1% (12.7–35.0%).

## Discussion

Compared with the non-pregnant reference ranges provided by manufacturers, serum TSH showed a downward trend during early pregnancy, with the upper limit decreasing by 21.7% and the lower limit decreasing by 85.7%. It maintained this descending trend in middle pregnancy, with the upper limit decreasing by 24.0% and the lower limit decreasing by 40.7%. Then, in late pregnancy, serum TSH gradually increased to non-pregnant levels. For serum FT4, the upper limit changed slightly, with the lower limit increasing by 6.8% compared to non-pregnant levels in early pregnancy. Then, serum FT4 gradually declined, with the upper limit decreasing by 21.8% and the lower limit decreasing by 12.7% in the second trimester. It kept decreasing in the third trimester, with the upper limit decreasing by 25.1% and the lower limit decreasing by 20.9%.

Pregnancy causes increases in renal iodine excretion, thyroxine binding proteins, and thyroid hormone production. A healthy thyroid adjusts thyroid hormone metabolism, iodine uptake, and the hypothalamic-pituitary-thyroid axis to mediate such changes. The peak rise in hCG also occurs during early pregnancy (13, 14). Maternal hCG plays a direct role in stimulating the TSH receptor to produce thyroid hormone, resulting in a decrease in serum TSH. Thus, serum hCG increases in association with a corresponding reduction in serum TSH (2, 3). Starting at gestational 6–8 weeks, maternal serum estrogens increase progressively until term, which is accompanied by total T4 increasing, FT4 decreasing, and TSH progressively increasing throughout the pregnancy (15). Therefore, the non-pregnant reference ranges for thyroid function tests are not applicable to pregnant women. National guidelines throughout the world have recommended the use of gestational- and population-specific serum TSH and FT4 reference ranges to diagnose thyroid disease during pregnancy (4, 5) (16–20). According to the 2017 ATA guidelines, 2.5 mU/L was no longer used as the serum TSH upper limit cut-off value to diagnose hypothyroidism in early pregnancy, and 4.0 mU/L was recommended when internal or transferable pregnancy-specific TSH reference intervals were unavailable (5). Since the serum TSH upper limit in the American general population is usually 4.5 mU/L, it generally decreased by 0.5 mU/L in the first trimester, resulting in the cut-off value of 4.0 mU/L (6).

Although the 2017 ATA guidelines provided a convenient and feasible method for determining the serum TSH upper limit in early pregnancy, whether 4.0 mU/L is suitable for pregnant Chinese women needs to be explored. First, serum TSH reference ranges vary among different ethnicities due to cultural, environmental, geographic and genetic factors (21–23). Second, sex differences exist in TSH circadian rhythms. Third, serum TSH values change throughout the 24-h cycle and progressively increase with age (12). Fourth, iodine is the main ingredient in the synthesis of thyroid hormones. Since the implementation of mandatory universal salt iodization in 1996, China has eliminated iodine deficiency and become an iodine-sufficient country (24). Epidemiological studies also found that the resident's average serum TSH level has risen due to the effects of increased iodine intake (25). A similar epidemiological survey reported by Korea showed that there was high iodine intake in Korea, resulting in serum TSH exhibiting a right-shifted distribution in that population (26).

TSH is regarded as one of the principal indicators to diagnose primary hyperthyroidism and hypothyroidism. Our study compared the gestational upper and lower limits for serum TSH with the non-pregnant reference intervals provided by the test manufacturers. We found that regardless the kind of kit or test method, the serum TSH upper limit decreased by ~22% and the lower limit decreased by ~85% in early pregnancy. What we found especially interesting was that the non-pregnant upper limit declined by 22% was very close to 4.0 mU/L. However, the difference between 4.0 mU/L and the non-pregnant TSH upper limit minus 0.5 mU/L, according to the 2017 ATA guideline's recommendation (5), was obvious. Although the difference between 4.0 mU/L and the real TSH upper limits of pregnant Chinese women cannot be eliminated, the difference was not significant. Our findings further suggest that if we use 4.0 mU/L as a sub-optimal approach to identify serum TSH upper limit in early pregnancy, this limit represents a relative descent rate in the non-pregnant TSH upper reference limit of 22% rather than a reduction of ~0.5 mU/L.

However, we must stress that the population of a local institute or laboratory and pregnancy-specific serum TSH reference ranges should optimally define the gestational-specific serum TSH reference range. If unavailable, pregnancy-specific TSH reference ranges obtained from similar patient populations and detected by similar test assays should be the alternatives. If the above two conditions are not available, 4.0 mU/L or the serum TSH upper limit, which is 22% lower than the non-pregnant level, may be used as a sub-optimal approach to identify the serum TSH reference ranges in pregnancy for diagnosing gestational thyroid diseases.

T4 is considered an important index for the diagnosis of overt gestational hypothyroidism and hypothyroxinemia. At present, serum FT4 is used as a diagnostic indicator for hypothyroidism and hypothyroxinemia in the majority of clinical laboratories. The 2017 ATA guidelines declared that the accuracy of detecting serum FT4 by indirect analog immunoassays was influenced by pregnancy and manufacturer diversity. Gestational- and method-specific serum FT4 reference ranges should be established, but they are difficult to implement (5). According to the studies we included, serum FT4 showed an upward trend in the first trimester compared to non-pregnant levels. The upper limit fluctuated slightly, while the lower limit increased by ~7.0%. Serum FT4 decreased in the second trimester, with the upper limit decreasing by ~20% and the lower limit decreasing by ~15%. Subsequently, serum FT4 declined more profoundly in the third trimester, with the upper limit decreasing by ~25% and the lower limit decreasing by ~20%. Thus, by comparing with the non-pregnant reference ranges provided by manufacturers or measurements in the local population, we can diagnose hypothyroxinemia once the serum FT4 lower limit decreases by more than 15% in middle pregnancy and 20% in late pregnancy.

Our analysis of the included studies found that the gestational TSH reference ranges are broader than those of the non-pregnant population, mainly because the serum TSH upper limit decreased less than the lower limit. One possible explanation for this phenomenon is that women with subclinical hypothyroidism have an impaired thyroidal response to hCG stimulation, and women with a lower thyroid functional capacity may already have high-normal TSH concentrations going into pregnancy (27). So, in the whole population, the TSH upper limit probably does not decrease steeply. The upper and lower limit of serum FT4 almost synchronously declined in pregnancy, resulting in no obvious change in the breadth of the reference range.

## Limitations

Our study had some limitations. We only included the studies from China, without considering other countries or ethnic groups. Our study represented the serum TSH and FT4 reference ranges of a pregnant Chinese population; due to the paucity of studies calculating good population-based reference ranges for non-pregnancy, we did not acquire accurate normal TSH and FT4 reference ranges, which can be seen as the gold standard for comparison (8). In addition, our meta-analysis only included kits published and meeting inclusion criteria. Kits such as the Bayer ASC 180, LIAISON, and TOSOH were not included because of few or no publications; A minimum of approximately 400 women is required, due to the high interindividual variability and skewness for TSH but also to some extent FT4 (9). In our meta-analysis, the number of women included in most of the studies was lower than 400.

## Conclusion

Our meta-analysis found that serum TSH decreased in the first and second trimesters of pregnancy and exhibited an upward trend to non-pregnant levels in the third trimester. Furthermore, serum FT4 increased slightly in the first trimester and decreased gradually in the second and third trimesters. The relative descent or ascent rate compared with the non-pregnant reference intervals may explain the change rules of gestational serum TSH and FT4. In the first trimester, using 4.0 mU/L as the cut-off point of the serum TSH upper limit is a sub-optimal approach for pregnant Chinese women. Generally, this limit represents a relative descent rate in the non-pregnant TSH upper reference limit of 22%.

## Author contributions

XG and YL: Conceived and designed the meta-analysis; XG, JL, and AL: Performed the meta-analysis; XG: Analyzed the data, wrote the manuscript, statistical analyses and paper writing; WS: Contributed material/analysis tools; XG and YL: Reference collection and data management; XG, ZS, and WT: Study design.

### Conflict of interest statement

The authors declare that the research was conducted in the absence of any commercial or financial relationships that could be construed as a potential conflict of interest.
