# Supplementary material for: Gestational TSH and FT4 Reference Intervals in Chinese Women: A Systematic Review and Meta-Analysis
Source: Front Endocrinol (Lausanne). 2018 Aug 3;9:432. doi: 10.3389/fendo.2018.00432 (PMC6086137; doi:10.3389/fendo.2018.00432)
Supplement: Supplementary file 1 [file Table_1.DOCX]

**Supplement table 1** The Newcastle-Ottawa quality assessment scale (NOS) was selected to assess the quality of the included studies by using the “star system”. Full score = 9 and the study which was graded greater than or equal to 6 stars could be considered as high-quality study ^(13)^.

| **Cohort Star Template** | | | | | | | | | | | | |
| --- | --- | --- | --- | --- | --- | --- | --- | --- | --- | --- | --- | --- |
| **Study** | **Manufacture** | **samples** | **Selection** | | | | **Comparability** | | **Outcome** | | | **Quality assessment** |
|  |  |  | **①** | **②** | **③** | **④** | **⑤** | | **⑥** | **⑦** | **⑧** |  |
| Li J, 2008 | DPC | 1118 | 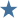 | 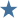 | 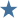 | 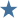 | 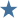 | 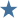 | 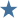 | 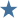 | 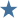 | 9 |
| Liu JH, 2016 | Roche/Beckman/Abbott | 947 | 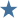 | 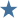 | 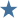 | 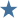 | 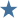 | 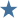 | 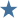 | 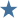 | 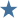 | 9 |
| Wang QW, 2011 | Roche | 1756 | 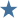 | 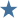 | 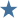 | 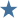 | 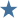 | 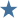 | 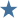 | 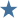 | 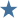 | 9 |
| Duan YF, 2015 | Bayer ADVIA Centaur | 3978 | 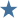 |  | 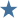 | 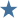 |  |  | 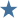 | 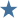 | 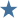 | 6 |
| Fan JX, 2013 | Roche/Abbott | 693 | 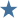 |  | 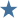 | 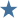 |  |  | 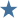 | 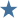 | 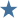 | 6 |
| Yan YQ, 2011 | Bayer ADVIA Centaur | 613 | 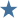 | 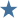 | 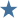 | 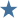 | 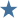 | 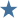 | 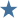 | 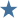 |  | 8 |
| Chen QQ, 2016 | Beckman | 281 | 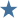 | 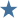 | 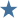 | 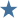 | 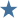 | 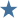 | 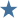 | 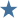 | 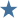 | 9 |
| Yu L, 2014 | Beckman | 877 | 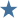 | 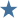 | 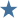 | 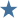 | 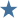 | 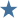 | 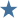 | 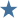 |  | 8 |
| Chen L, 2016 | Beckman | 526 | 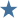 | 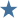 | 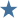 | 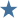 | 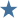 | 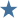 | 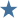 | 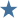 | 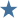 | 9 |
| Li CY, 2014 | Roche | 640 | 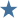 | 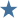 | 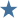 | 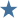 | 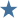 | 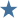 | 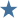 | 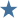 | 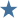 | 9 |
| Fan JX, 2015 | Roche/Abbott/Bayer/DPC | 200 | 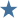 | 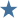 | 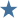 | 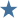 | 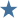 | 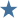 | 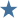 | 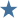 | 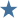 | 9 |
