# Supplementary material for: Gestational TSH and FT4 Reference Intervals in Chinese Women: A Systematic Review and Meta-Analysis
Source: Front Endocrinol (Lausanne). 2018 Aug 3;9:432. doi: 10.3389/fendo.2018.00432 (PMC6086137; doi:10.3389/fendo.2018.00432)
Supplement: Supplementary file 2 [file Table_2.DOCX]

**Supplement table 2 Stratified meta-analysis of relative descent or ascent rate among 5 kinds of kits**

| **Subgroups** | **Relative descent or ascent rate % (95%CI)** | **No of studies** | **Heterogeneity** | |
| --- | --- | --- | --- | --- |
|  |  |  | **I^2^%** | **p Value** |
| **^a^ TSH, T1, 97.5^th^** | Summary: 21.7% (20.4%-23.1%) | Summary: 11 | Summary: 89.03 | Summary: <0.05 |
| Roche | 22.7% (20.4%-25.0%) | 4 | 29.59 | 0.235 |
| Bayer | 18.3% (16.1%-20.7%) | 2 | 95.22 | <0.05 |
| Abbott | 24.8% (20.2%-30.2%) | 2 | - | 0.364 |
| DPC | 17.6% (13.9%-22.1%) | 2 | 96.74 | <0.05 |
| Beckman | 25.5% (22.3%-28.9%) | 3 | 94.10 | <0.05 |
| **^a^ TSH, T1, 2.5^th^** | Summary: 85.7% (84.5%-86.8%) | Summary: 11 | Summary: 90.24 | Summary: <0.05 |
| Roche | 87.6% (85.5%-89.4%) | 4 | 87.10 | <0.05 |
| Bayer | 91.4% (89.7%-92.9%) | 2 | 82.93 | <0.05 |
| Abbott | 91.4% (87.6%-94.2%) | 2 | - | 1 |
| DPC | 78.6% (74.5%-82.1%) | 2 | - | 0.521 |
| Beckman | 79.8% (76.6%-82.6%) | 3 | 86.81 | <0.05 |
| **^b^ FT4, T1, 2.5^th^** | Summary: 6.8% (5.9%-7.7%) | Summary: 9 | Summary: 79.06 | Summary: <0.05 |
| Roche | 5.4% (4.1%-7.2%) | 3 | 87.86 | <0.05 |
| Bayer | 6.6% (5.3%-8.3%) | 2 | 76.97 | <0.05 |
| Abbott | 3.4% (1.8%-6.3%) | 2 | 62.63 | 0.102 |
| DPC | 4.4% (2.4%-7.7%) | 1 | - | 1 |
| Beckman | 9.4% (7.5%-11.7%) | 3 | 70.40 | <0.05 |
| **^b^ FT4, T2, 97.5^th^** | Summary: 21.8% (20.3%-23.5%) | Summary: 8 | Summary: 95.84 | Summary: <0.05 |
| Roche | 20.3% (17.3%-24.2%) | 2 | 92.68 | <0.05 |
| Bayer | 30.5% (27.9%-33.3%) | 2 | 82.67 | <0.05 |
| Abbott | 12.6% (9.7%-16.3%) | 2 | 95.83 | <0.05 |
| DPC | 5.5% (3.6%-8.3%) | 1 | - | 1 |
| Beckman | 12.6% (10.1-15.3%) | 3 | 92.53 | <0.05 |
| **^b^ FT4, T2, 2.5^th^** | Summary: 12.7% (11.5%-14.0%) | Summary: 8 | Summary: 92.05 | Summary: <0.05 |
| Roche | 16.7% (13.6%-20.2%) | 2 | 58.23 | 0.122 |
| Bayer | 5.1% (3.9%-6.5%) | 2 | 70.75 | 0.064 |
| Abbott | 17.7% (14.6%-21.3%) | 2 | 55.73 | 0.133 |
| DPC | 2.6% (1.4%-4.8%) | 1 | - | 1 |
| Beckman | 15.2% (12.7%-17.9%) | 3 | - | 0.437 |
| **^b^ FT4, T3, 97.5^th^** | Summary: 25.1% (23.6%-26.7%) | Summary: 8 | Summary: 83.72 | Summary: <0.05 |
| Roche | 32.0% (28.2%-36.1%) | 2 | 24.51 | 0.250 |
| Bayer | 28.1% (25.3%-31.0%) | 2 | - | 0.603 |
| Abbott | 22.3% (18.9%-26.0%) | 2 | - | 0.372 |
| DPC | 19.8% (16.0%-24.2%) | 1 | - | 1 |
| Beckman | 19.2% (16.5%-22.3%) | 3 | 86.27 | <0.05 |
| **^b^ FT4, T3, 2.5^th^** | Summary: 20.9% (14.8%-27.3%) | Summary: 8 | Summary: 74.45 | Summary: <0.05 |
| Roche | 25.1% (21.6%-29.0%) | 2 | 60.17 | 0.113 |
| Bayer | 17.8% (15.5%-20.4%） | 2 | - | 0.411 |
| Abbott | 24.2%（20.8%-28.0%） | 2 | - | 0.339 |
| DPC | 14.8% 11.5%-18.8%) | 1 | - | 1 |
| Beckman | 21.4% (18.6%-24.6%） | 3 | 76.75 | 0.014 |

*T1:* the first trimester of pregnancy; *T2:* the second trimester of pregnancy; *T3:* the third trimester of pregnancy; *TSH:* thyroid stimulating hormone; *FT4:* free T4.

^a^ The subgroup analysis about relative descent rate of serum TSH upper (97.5^th^) and lower (2.5^th^) limits in the first trimester of pregnancy.

^b^ The subgroup analysis about relative ascent rate of serum FT4 lower (2.5^th^) limit in the first trimester and relative descent rate of serum FT4 upper (97.5^th^) and lower (2.5^th^) limits in the second and third trimesters of pregnancy.
